# Factors associated with the identification of drug therapy problems among older patients in Primary Health Care

**DOI:** 10.31744/einstein_journal/2022AO6544

**Published:** 2022-03-29

**Authors:** Bianca Menezes Dias, Djenane Ramalho-de-Oliveira, Bruna Damazio Santos, Carina de Morais Neves, Grazielli Cristina Batista de Oliveira, Daniela Álvares Machado Silva, Yone de Almeida Nascimento, Annaline Stiegert Cid, Gabriela Oliveira Buzelin, Sabrina Gonçalves Ferreira, Kirla Barbosa Detoni, Mariana Martins Gonzaga do Nascimento

**Affiliations:** 1 Faculdade de Farmácia Universidade Federal de Minas Gerais Belo Horizonte MG Brazil Faculdade de Farmácia, Universidade Federal de Minas Gerais, Belo Horizonte, MG, Brazil.; 2 Maternidade & Hospital Octaviano Neves Belo Horizonte MG Brazil Maternidade & Hospital Octaviano Neves, Belo Horizonte, MG, Brazil.; 3 Prefeitura Municipal de Lagoa Santa Lagoa Santa MG Brazil Prefeitura Municipal de Lagoa Santa, Lagoa Santa, MG, Brazil.; 4 Secretaria Municipal de Saúde de Belo Horizonte Belo Horizonte MG Brazil Secretaria Municipal de Saúde de Belo Horizonte, Belo Horizonte, MG, Brazil.; 5 Faculdade Pitágoras Betim MG Brazil Faculdade Pitágoras, Betim, MG, Brazil.

**Keywords:** Aged, Primary Health Care, Pharmaceutical services, Medication therapy management, Polypharmacy

## Abstract

**Objective:**

To determine the frequency of drug therapy problems among older adults in Primary Health Care, and to analyze the factors associated with their identification in the initial patient assessment, carried out by pharmacists offering medication therapy management services.

**Methods:**

A cross-sectional study conducted with data from 758 older adults followed up in medication therapy management services in Primary Health Care in the cities of Belo Horizonte, Betim, and Lagoa Santa (MG, Brazil). Univariate and multivariate analyses were performed to evaluate the factors associated with identification of four or more drug therapy problems in the initial clinical assessment.

**Results:**

A total of 1,683 drug therapy problems were identified, 73.6% of older patients had at least one problem. The most frequent problems were nonadherence (23.0%) and the need for additional drug therapy (18.0%). Polypharmacy, chronic obstructive pulmonary disease, hypertension, *diabetes mellitus*, heart failure, and aged 75 years or older remained positively and statistically associated with identification of four or more drug therapy problems (p<0.05).

**Conclusion:**

There is a high frequency of problems related to medication use among older users of Primary Health Care, and the medication therapy management services should be prioritized to the older patients, who present with polypharmacy, chronic obstructive pulmonary disease, hypertension, *diabetes mellitus*, heart failure, and age ≥ 75 years, since they are more likely to have more drug therapy problems.

## INTRODUCTION

The use of multiple medications is a reality among older patients in Primary Health Care (PHC) of the Brazilian Public Health System (SUS - *Sistema Único de Saúde).* Although it can be clinically indicated for several patients, polypharmacy, defined as “the use of five or more medications,”^(1)^ has been associated with negative health outcomes, especially for older adults, due to pharmacokinetic and pharmacodynamic changes resulting from aging.^(2-4)^

This context indicates the social need for prevention, identification, and resolution of drug therapy problems (DTP), a priority among older adults. Drug therapy problems are defined as unwanted events experienced by patients, which involve or suspect involvement in the use of medications and interfere in the expected results of therapy, requiring professional judgment to be solved.^(5)^ This demand can be met through the provision of medication therapy management (MTM) services, based on the theoretical and methodological framework of Pharmaceutical Care in Brazil. This patient-centered practice follows a structured and systematized clinical method of decision-making in pharmacotherapy, whose objective is to ensure patients use the most indicated, effective, safe, and convenient medications for their health conditions, and achieve the best possible health outcomes.^(6)^

In PHC, the MTM has demonstrated positive clinical impacts,^(7-9)^ and its provision is relevant in this setting, playing a central role in the coordination of actions and services within the proposed model of Comprehensive Health Care for the Elderly, discussed in the XXX National Congress of Municipal Health Departments.^(10)^It is important to highlight, however, that since about 75% of Brazilian older adults are cared for exclusively through the SUS, the new reality of population aging demands more cost-effective health care models, with selection criteria that go beyond age. This significant increase in the number of older adults in Brazil puts pressure on health systems to include the specificity and heterogeneity of the aging process, and to design new strategies to prioritize care.^(10,11)^

In the MTM services offered to the older population, risk stratification becomes even more necessary due to the shortage of pharmacists in the public sector, in addition to other difficulties faced by these professionals, such as lack of structural support for the work, high population coverage, and the need to dedicate part of their workload to management activities in the Primary Care Units (PCU).^(12)^

## OBJECTIVE

To determine the frequency of drug therapy problems among older patients at Primary Health Care, and to analyze the factors associated with their identification in the initial assessment by pharmacists providing medication therapy management services.

## METHODS

### Study design

This is a cross-sectional study conducted with older adults followed up in MTM services at PHC.

### Location and description of the drug therapy management service

A study performed with data regarding the MTM services implemented in the PHC of the cities of Belo Horizonte, Betim, and Lagoa Santa (MG). In Belo Horizonte, the MTM services were provided from February 2015 to February 2017 by a pharmacist linked to the *Núcleo Ampliado de Saúde da Família e Atenção Básica* (NASF-AB) [Extended Family Health and Primary Care Center]. In Betim, the service was offered from September 2015 to March 2016 by eight pharmacists hired by the city government. In Lagoa Santa, MTM consultations were provided from July 2014 to July 2017 by five pharmacists hired by the municipality. These pharmacists received 60 hours of training by MTM specialists prior to offering the service, as well as mentoring during the initial phases of implementation.

In the MTM services considered, the theoretical and methodological framework of Pharmaceutical Care and the Pharmacotherapy Workup decision-making process, proposed by Cipolle et al.,^(6)^ were used. In this process, the pharmacist analyzes all the patient’s health conditions and the drugs in use (prescribed, non-prescribed, and use of traditional therapies), with the aim of preventing, identifying, and solving the DTP and achieving desirable health outcomes.^(5,6)^In this context, at each patient encounter, the clinical pharmacist performs a complete evaluation of the pharmacotherapy following the Pharmacotherapy Workup, documenting in detail in medical records the patient’s health conditions, the effectiveness and safety parameters, and the evolution of the patients and the evaluated DTP.

Drug therapy problems are classified according to the evaluation of indication, effectiveness, safety, and adherence to each medication used by the patient. Thus, regarding the indication, the drug may be unnecessary (DTP 1) or there may be a need for an additional drug (DTP 2); regarding effectiveness, it is considered that the drug may not be effective, requiring an exchange (DTP 3), or the dose may be low (DTP 4); for safety, the patient may be experiencing an adverse reaction, requiring a change of the medication (DTP 5), or the medication may be at a high dose (DTP 6); and, as for adherence, the patient may not be appropriately complying with the treatment (DTP 7). The main causes of non-adherence by the patient include difficulty in understanding the orientation provided by health professionals, financial difficulty in buying the drug, or its unavailability at the pharmacy or drugstore, patient prefers not to take it, forgets to take it, or has difficulty in administering the drug (*e.g.,* difficulty in swallowing a very large tablet).^(6)^

In the MTM, the evaluation of pharmacotherapy occurs on an individual basis and according to the clinical response of each patient. Thus, the identification of DTP is clinical and not theoretical, and for this reason, DTP 4, low dose, and DTP 6, high dose, are not synonymous of “underdose” or “overdose”, in relation to the dose recommended by the literature, but correspond to cases in which the dose is low or high for achieving the therapeutic objectives for each health problem. Thus, according to the adopted theoretical framework,^(6)^high-dose DTP are considered the undesirable events that are resolved by reducing the administered daily dose, including the “overdose” itself.

Thus, to identify the DTP 4 and DTP 6, an evaluation of the individual clinical response of the older patient was performed, according to the parameters of effectiveness and safety.

After identifying the DTP, the pharmacist performs interventions with the prescriber or patient in order to solve it.^(5,6)^ In this study, the whole care process was documented in a standardized way in a medical record of the MTM service.

### Study population

The study population included all older patients (individuals aged 60 years or older, as established by the World Health Organization (WHO) for developing countries) seen at the MTM services in PHC in the analyzed municipalities. For inclusion in the MTM services, no specific criteria were adopted.

### Data collection and study variables

Data collection was performed directly from the medical records of the service by three researchers with MTM experience and stored in the Stata software, version 12. All data collection was coordinated by a researcher who validated the data obtained by double-checking the sum of the variables with a fourth researcher.

In this study, the dependent variable was the number of DTP in the pharmacists’ initial assessment, dichotomized according to its third quartile (75%) and defined as the identification of four or more DTP in the initial assessment. This cutoff point at the third quartile was intended to flag patients with a higher number of DTP and who, therefore, had a higher potential risk related to their pharmacotherapy, and should be prioritized for inclusion in the MTM services. In the present investigation, the “initial assessment” considered the patient’s first two visits to the MTM service, to ensure all the patient’s health conditions and medications in use were properly evaluated. For this scenario, this methodological definition was important, since patients were often referred to the pharmacist right after the end of the visit with the physician, and many times, they did not take all their prescriptions, exams, and medication packs with them at the first MTM visit.

The independent variables were sociodemographic (sex and age), clinical (number and types of health conditions documented in the MTM medical record) and pharmacotherapeutic (number of medications used in the initial assessment). Numerical variables were also dichotomized according to the median, to facilitate the identification of priority criteria for care in MTM services. This study adopted the definition of polypharmacy as “the use of five or more medications.”^(1)^

### Data analysis

The collected information was entered into the Excel^®^ program and later transferred and organized in a database in the Stata software version 12 (Stata Corp. College Station, United States). The latter software was used for all statistical analyses. Initially, a descriptive analysis was performed, determining absolute and relative frequencies for categorical variables, and numerical variables were described as mean and standard deviation (SD), or median and interquartile range (IQR).

The evaluation of the association between independent variables and the identification of four or more DTP in the initial assessment (dependent variable) was initially performed through univariate analysis, according to Pearson’s χ^2^ test. Later, considering the absence in the literature of previous investigation of associated factors in the scenario proposed in this study, it was decided to adopt a parsimonious multivariate model, and all variables were included in the final model, regardless of any statistical criteria. In the multivariate analysis, we used the stepwise logistic regression method, which has automatic deletion and keeps in the final model only the variables with statistical adequacy. The adequacy of the multivariate model was assessed by the Hosmer-Lemeshow test. Both univariate and multivariate analyses were based on the odds ratio (OR) result and its respective 95% confidence interval (95%CI). The statistical significance level adopted in the study was 5%.

### Ethical aspects

This study is part of the project Clinical, Economic, Humanistic, Cultural, and Educational Outcomes in MTM at SUS, approved by the Ethics and Research Committee of the *Universidade Federal de Minas Gerais* (UFMG), registration CAAE: 25780314.4.0000.5149 and opinion #3.060.175. The study was exempted from the Informed Consent Form.

## RESULTS

We used data from 758 older adults seen at the MTM services, with the majority being female (446; 58.8%). The sample had a mean age equal to 70.2±7.7 years (minimum of 60 years and maximum of 98 years). The mean number of health problems in the initial evaluations was 3.8±1.9 (minimum of zero and maximum of 11), and the most prevalent diseases were hypertension (HTN) (669; 88.3%), dyslipidemia (382; 50.4%), and *diabetes mellitus* (DM) (346; 45.7%). At initial assessments, 63.5% of the older adults (n=481) were taking five or more medications, with a mean of 5.8±3.1 medications per patient (minimum of zero and maximum of 18) (Table 1).

**Table 1:** Sociodemographic, clinical, and pharmacotherapeutic characteristics of the sample of 758 older adults assisted in medication therapy management services

Characteristics	
Sociodemographic	
Female sex	446 (58.8)
Age, years	70.2±7.7
Clinical	
Number of health problems at the initial assessment*	3.8±1.9
Comorbidities	
HTN	669 (88.3)
Dyslipidemia	382 (50.4)
DM	346 (45.7)
Central nervous system diseases (except dementia and other cognitive disorders)	141 (18.6)
Dementia	7 (0.92)
HF	75 (9.9)
Pharmacotherapeutic	
Number of medications per patients at the initial assessment*	5.8±3.1
Number of DTP at the initial assessment*	2.2±2.2

* Initial assessment: first and second visits for drug therapy management.

Results expressed by n (%) or mean ± standard deviation.

HTN: hypertension; DM: *diabetes mellitus*; HF: heart failure; DTP: drug therapy problems.

A total of 1,683 DTP were identified in the initial assessments, and 73.6% of older patients (n=558) had at least one DTP, with a mean number of DTP equal to 2.2±2.2 per patient (minimum of zero and maximum of 12). Among the total number of identified DTP, the most frequent were non-adherence (23.0%) and need for additional medication (18.0%) (Table 2).

**Table 2:** Drug therapy problems stratified by category

DTP Category	
Unnecessary medication	286 (17.0)
Need for additional medication	304 (18.0)
Non-effective medication	101 (6.0)
Low dose	293 (17.4)
Adverse drug reaction	168 (10.0)
High dose	144 (8.6)
Non-adherence	387 (23.0)
Total	1,683 (100.0)

Results expressed by n (%).

DTP: drug therapy problem.

Table 3 shows the results of the univariate and multivariate analyses of the factors associated with the identification of four or more DTP in the initial evaluations. Use of five or more medications, presence of chronic obstructive pulmonary disease (COPD), HTN, DM, and heart failure, and age 75 years or older remained positively and statistically significantly associated, with the identification of four or more DTP in the initial evaluations of the MTM services (p<0.05).

**Table 3:** Univariate and multivariate analysis of factors associated with the identification of four or more drug therapy problems at initial assessment*

Variables	Univariate analysis		Multivariate analysis	
	
	OR (95%CI)^**#**^	p value^†^	OR (95%CI)^**#**^	p value^‡^
Sociodemographic				
Male sex	0.73 (0.51-1.03)	0.074		
Age, completed years				
60-74	1.0	-	1.0	-
≥75	1.55 (1.08-2.23)	0.018	1.70 (1.14-2.53)	0.009
Clinical				
DM				
Yes	2.76 (1.94-3.91)	<0.001	2.02 (1.37-2.98)	<0.001
No	1.0	-	1.0	-
HTN				
Yes	4.02 (1.82-8.87)	0.001	2.56 (1.11-5.89)	0.028
No	1.0	-	1.0	-
Dyslipidemia				
Yes	1.62 (1.15-2.28)	0.005		
No	1.0	-		
Acute myocardial infarct				
Yes	1.25 (0.61-2.56)	0.54		
No	1.0	-		
HF				
Yes	2.91 (1.78-4.76)	<0.001	2.01 (1.19-3.40)	0.009
No	1.0	-	1.0	-
Dementia				
Yes	4.42 (0.98-19.94)	0.053		
No	1.0	-		
COPD				
Yes	3.44 (1.56-7.56)	0.002	3.38 (1.41-8.10)	0.006
No	1.0	-	1.0	-
Central nervous system disease (except dementias and other cognitive disorders)				
Yes	1.78 (1.19-2.66)	0.005	1.55 (0.99-2.40)	0.052
No	1.0	-	1.0	-
Pharmacotherapeutical				
Number of medications used at the initial assessment^§^				
0-4	1.0	-	1.0	-
≥5	7.17 (4.29-11.99)	<0.001	4.33 (2.49-7.53)	<0.001

* Estimated by logistic regression; ^†^ estimated according to Pearson’s χ^2^ test; ^‡^ estimated by stepwise logistic regression; statistically significant when <0.05; ^§^ initial assessment: first and second drug therapy management visit; ^#^ OR (95%CI): estimated by stepwise logistic regression.

OR: odds ratio; 95%CI: 95% confidence interval; DM: *diabetes mellitus*; HTN: hypertension; HF: heart failure; COPD: chronic obstructive pulmonary disease.

## DISCUSSION

The present study revealed a high number of DTP (1,683; mean of 2.2 DTP per patient) among the older patients followed-up in MTM services at PHC. Although no studies were identified about the provision of MTM specifically to the older patients, and that used the theoretical and methodological framework of Pharmaceutical Care for comparison, the frequency of identification of at least one DTP among patients included in this study (73.6%) was higher than that found by Strand et al.,^(13)^(61%), in a previous large study conducted in a Primary Care setting in the United States. This high prevalence of DTP shows that, although PHC has a structure designed to allow a continuous monitoring of patients within their cultural and socioeconomic context, in addition to care coordination and reduction of unnecessary use of specialized services, a high percentage of older users have non-optimized pharmacotherapy, which highlights the need for implementation of MTM services for this population.

However, in a context of population aging, shortage of pharmaceutical professionals to care for this population, and limited resources, it is crucial to define which elders would most benefit from these MTM services.^(11,12)^Therefore, risk stratification becomes fundamental to establish a comprehensive care network for the older patients in health systems, and older adults with health conditions associated with the occurrence of adverse events need priority access at SUS.^(10)^ In the present study, the identification of a high percentage of older patients followed-up in MTM services, at PHC, with at least one DTP (73.6%) shows the demand for determining which of these patients should be given priorty for inclusion in the MTM services, emphasizing the relevance of this study.

The most frequent DTP identified in this study in the initial visits was non-adherence to treatment (23.0%), followed by the need for additional medication (18%), and low dose (17.4%). In an international study that evaluated the clinical, economic and humanistic outcomes of the MTM over a long period (25 years), and in multiple visits, the most frequently identified DTP were need for additional medication, followed by low dose and no adherence to treatment.^(13)^In the present study, one justification for the DTP of non-adherence being the most frequent may be related to difficult access to medication. In a previous study to assess the availability of essential drugs at PHC of SUS, only 52.9% of drugs were available; and in most cases when the prescribed drugs were not available (75.6%), the dispensing unit did not provide guidance to the user.^(14)^ Thus, excluding this cause, the most frequent DTP in this study become similar to those from American studies previously described.^(13)^

As for the factors associated with the identification of a higher number of DTP in older users of PHC, the use of five or more medications was the most strongly associated variable. According to previous studies, polypharmacy in the older patients is associated with higher risk of using potentially inappropriate drugs, high anticholinergic burden of pharmacotherapy, adverse drug reactions, clinically relevant drug interactions, and poor compliance.^(15-17)^ This contributes to functional decline, hospitalizations, and death of the older adult, and the instruments used to screen for frailty include the number of medications on use as a criterion.^(3,16)^ This scenario underscores the need to include older adults using multiple medications in the MTM services, to prevent negative health outcomes and contribute to maintaining the patient’s autonomy and independence.

The second variable most strongly associated with the identification of four or more DTP in the initial visits of the older participants in the study was the presence of COPD. In the management of this disease, clinical guidelines are frequently updated, the use of multiple medications is prevalent, and the form of medication administration is complex. The inappropriate use of inhalers by the older adults is very frequent, mainly in those with cognitive impairment – and such factors may contribute to occurrence of DTP.^(18-20)^

In addition to polypharmacy and COPD, HTN and DM were also positively and independently associated with a higher occurrence of DTP in older users of PHC. In the older patients, HTN and DM are usually accompanied by cardiovascular complications and other comorbidities, and the use of multiple medications is frequent.^(21)^ Moreover, although these comorbidities contribute to high cardiovascular risk in older adults, potentiating the benefits of drug therapy in reducing morbidity and mortality, these patients are at increased risk of orthostatic hypotension, falls, and severe hypoglycemia resulting from inadequate antihypertensive and hypoglycemic therapy, respectively, which highlights the importance of individualization of pharmacotherapy in older adults.^(22-24)^ Another factor to be considered is the greater ease of the pharmacist in identifying DTP in older adultswith HTN and DM, since, due to the high prevalence of these comorbidities in the Brazilian population, a more extensive approach on their pharmacotherapeutic management is expected in undergraduate pharmacy courses.

As for heart failure, which was also positively associated with the dependent variable, it is known that it medical management is complex, and basic drug therapy involves the use of at least three drugs from different classes, in addition to the drugs used for frequently associated comorbidities.^(25)^

This favors the identification of DTP in the older patient with heart failure, especially in the context of low adherence of professionals to clinical guidelines. In a qualitative review conducted with data from Primary Care in different countries, it was found that most physicians were not familiar with the guidelines for diagnosis and management of heart failure, especially when the patient was older and with multiple morbidities and polypharmacy. Some physicians kept patients on diuretics only, possibly without knowing about the potential benefits of angiotensin-converting enzyme inhibitors and beta-blockers.^(26)^ Besides being indicated only for the advanced stages of heart failure and management of clinical decompensation events, loop diuretics in older adults are associated with hypotension, urinary incontinence, hyponatremia, and kidney injury.^(22,27)^

Finally, it was observed that advanced age (equal to or greater than 75 years) was also positively associated with a higher number of DTP. The pharmacokinetic and pharmacodynamic changes intrinsic to the aging process favor the occurrence of adverse events related to the use of medications. There is, for example, a higher percentage of body fat with a consequent increase in the half-life of lipophilic drugs, such as benzodiazepines, as well as a reduction in the levels of the neurotransmitter acetylcholine, with greater sensitivity to drugs with an anticholinergic burden. Consequently, older people are at even greater risk of sedation, falls, mental confusion, xerostomia, constipation, and urinary retention.^(17,28)^

As pointed out by Cipolle et al.,^(6)^ to establish which patients would most benefit from the MTM services is a difficult question to be answered, for reasons such as high prevalence of DTP, lack of sufficient data in medical records, and significant pressure from managers to include cost-effective services. This underscores the importance of the present study in delineating which older adults should be given priority for inclusion in MTM services at PHC, in the context of population aging and the need for optimization of health care resources. Moreover, it is worth noting that all pharmacists participating in the study, received training by specialists in MTM, reinforcing the importance of adequate training in relation to the clinical method, for standardization and quality assurance of the service provided. However, a limitation of this research is the short time of experience of the pharmacists responsible for the MTM consultations, regarding the clinical care of the older patient during the period considered, which may have underestimated the identification of DTP in these patients. In addition, the descriptive aspect of the present study is limited to the frequency of the identified DTP in the first visits, without specifying the main medications involved in them. It is suggested they will be contemplated in future studies that aim to explore more broadly the clinical aspects relevant to the identification, prevention, and resolution of DTP in the geriatric population.

The cross-sectional design of the study also has inherent limitations, such as no evaluation of the incidence of the variable that is the focus of the study DTP, allowing only bidirectional analysis between dependent, and independent variables. However, it has a pharmacoepidemiological design that meets well the objective of evaluating associated factors in a scenario that requires prioritization of patients, since there are no longitudinal studies elucidating such aspects.

## CONCLUSION

The frequency of drug therapy problems among older users of Primary Health Care was high. Older adults with polypharmacy, chronic obstructive pulmonary disease, hypertension, *diabetes mellitus*, heart failure, and/or advanced age should be prioritized for inclusion in medication therapy management services, since they are more likely to present a higher number of drug therapy problems.
